# Dipyridamole and adenosinergic pathway in Covid-19: a juice or holy grail

**DOI:** 10.1186/s43042-022-00354-1

**Published:** 2022-09-23

**Authors:** Hayder M. Al-kuraishy, Ali I. Al-Gareeb, Engy Elekhnawy, Gaber El-Saber Batiha

**Affiliations:** 1 Department of Pharmacology, Toxicology and Medicine, College of Medicine, Al-Mustansiriyah University, Baghdad, 14132 Iraq; 2 Pharmaceutical Microbiology Department, Faculty of Pharmacy, Tanta University, Tanta, 31527 Egypt; 3 Department of Pharmacology and Therapeutics, Faculty of Veterinary Medicine, Damanhour University, Damanhour, 22511 Al Beheira Egypt

**Keywords:** ALI, Adenosine, ARDS, SARS-CoV-2, NLRP3, Phosphodiesterase

## Abstract

**Background:**

Coronavirus disease 2019 (Covid-19) is an infectious worldwide pandemic triggered by severe acute respiratory coronavirus 2 (SARS-CoV-2). This pandemic disease can lead to pro-inflammatory activation with associated acute lung injury and acute respiratory distress syndrome.

**Main body of the abstract:**

SARS-CoV-2 infection is linked with inhibition of adenosine and activation of phosphodiesterase. Dipyridamole (DIP) is a nucleoside transport and phosphodiesterase inhibitor so that it may potentially affect SARS-CoV-2 infection and its accompanying inflammations. Therefore, the primary objective of this mini-review study was to elucidate the potential beneficial impacts of DIP on the adenosinergic pathway in Covid-19. A systemic search was done using online databases with relevant keywords. The findings of the present study illustrated that DIP directly or indirectly, through augmentation of adenosine and inhibition of phosphodiesterase, mitigates Covid-19 outcomes.

**Conclusion:**

Our study concluded that DIP has a potential therapeutic effect in the management and treatment of Covid-19. This could be attained either directly, through anti-SARS-CoV-2, anti-inflammatory, and anti-platelets properties, or indirectly, through augmentation of extracellular adenosine, which has anti-inflammatory and immune-regulatory effects. However, extensive randomized clinical trials, and clinical and prospective research in this area are required to demonstrate the safety and therapeutic efficacy of DIP and adenosine modulators in the treatment of Covid-19.

## Background

Coronavirus disease 2019 (Covid-19) is a recent worldwide infection that was recognized for the first time in late December 2019 in Wuhan, China [1], triggered by severe acute respiratory coronavirus 2 (SARS-CoV-2). This disease may lead to critical instabilities systemically, including pro-inflammatory activation, cytokine storm, and associated damage to various organs [2]. Covid-19 affects various body systems, predominantly the respiratory system. The main presentation of the disease is acute lung injury (ALI) and acute respiratory distress syndrome (ARDS). Besides, acute kidney, pancreatic, and cardiac injury, neurological disorders, and endothelial dysfunction might occur as extra-pulmonary manifestations [3]. These multiple impacts of Covid-19 are attributed to the presence of angiotensin-converting enzyme 2 (ACE2), a receptor for SARS-CoV-2, on the cells of multiple organs, which facilitates its entry into various host cells [3, 4]. ACE2 receptor is principally articulated in the lung alveolar cells type II and proximal renal tubules. When SARS-CoV-2 binds to the ACE2, these defending receptors will be down-regulated. Consequently, the level of vasoconstrictors angiotensin II (Ang II) would increase, and the vasodilator angiotensin (Ang 1–7) (Ang 1–9) would decrease, accompanied by the production of the pro-inflammatory cytokines [5].

Dipyridamole (DIP) is a nucleoside transport and phosphodiesterase (PDE) inhibitor that is used as an antiplatelet agent [6]. The primary mechanisms of DIP are inhibition of adenosine reuptake in the red blood cells (RBCs), platelets, and endothelial cells, as well as inhibition of PDE. DIP has diverse clinical effects. It reduces pulmonary hypertension, improves coronary blood flow, myocardial function and perfusion, mild peripheral vasodilator effect, and endothelial functions by inhibiting the discharge of the pro-inflammatory cytokines and preventing the sub-endothelial thrombogenicity.

As well, SARS-CoV-2 infection is usually interrelated with inhibition of adenosine (AD) as well as activation of PDE [7]. Since Covid-19 is linked with cardiovascular complications and coagulopathy, the main target of our mini-review study was to elucidate the potential impacts of DIP on the adenosinergic pathway in Covid-19.

## Adenosinergic pathway and DIP in Covid-19

It is known that AD is a primary nucleoside for building RNA and DNA, and it has different derivatives, including adenosine monophosphate (AMP), adenosine diphosphate (ADP), and adenosine triphosphate (ATP). These derivatives act as signal transductions for the modulation of different physiological processes. AD acts on the specific receptors subtypes, including A_1_, A_2A_, A_2B_, and A_3,_ broadly found in different tissues. It acts as a cytoprotective signal against tissue injury. AD has immunosuppressive and anti-inflammatory effects (A_2A_, A_2B_) and is upregulated during ischemia and tissue hypoxia [8].

Cellular AD concentration is controlled by specific regulators, which are adenosine kinase (ADK), adenosine deaminase (ADA), and equilibrative nucleoside transporter-1 (ENT-1). ADA metabolizes AD to inosine when AD is present at a higher concentration owing to its low binding capacity. At the same time, ADK metabolizes AD, at baseline concentration, to 5-inosine monophosphate due to its higher affinity and capacity [9, 10]. In addition, ADA regulates the expression of ADA-binding protein (ADA-BP) on CD26 and dipeptidyl dipeptidase 4 (DPP-4). Interestingly, besides to ACE2 receptor, which has a low expression in the lung, ADA-BP of DPP-4/CD26 is considered as a potential receptor for binding and entrance of SARS-CoV-2. Thus, ADA competes with SARS-CoV-2 for binding to ADA-BP. Moreover, ADA activators like pegademase ADA or recombinant ADA have been effectively used in the management of the human immunodeficiency virus (HIV) [11]. It has been proposed that ADA regulates and fine-tunes AD's immunosuppressive and anti-inflammatory effects. Besides, the early administration of recombinant ADA attenuates the binding of Middle East respiratory syndrome coronavirus (MERS-CoV) to its entry point, the DPP-4 receptor [12]. Since there is a 50% similarity in the genome sequence between SARS-CoV-2 and MERS-CoV, the recombinant ADA may minimize the severity of Covid-19 by inhibiting the binding between SARS-CoV-2 and DPP-4 [13]. Therefore, DPP-4 inhibitors may diminish SARS-CoV-2 pathogenesis and Covid-19 severity in diabetic people via modulation of SARS-CoV-2 entry and the accompanied inflammations [14]. Expressions of DPP-4 receptors are higher in patients with diabetes mellitus, nicotine smoking, chronic obstructive pulmonary disease (COPD), and obesity. This issue might explain the susceptibility of Covid-19 patients to the development of ALI and ARDS [15].

DIP doesn't affect ADA activity or expression of ADA-BP; however, a higher intra-lymphocytic concentration is linked with immune suppression in patients with chronic kidney diseases. Also, high interferon-gamma (INF-γ) activates ADA activity in patients with HIV, thus, both INF-γ and ADA are regarded as prognostic and diagnostic factors for disease severity [16]. Tan et al. [17] experimental study demonstrated that DIP reduces the discharge of the pro-inflammatory cytokines and activation of T cells via modulation of the AD pathway. Indeed, ADA activity is negatively correlated with DPP-4 activity [17]. Therefore, AD elevation by DIP may increase ADA activity, reducing DPP-4 expression and interaction with SARS-CoV-2. However, ADA might be a possible target for SARS-CoV-2, causing a significant reduction in the cellular concentration of AD and the development of ALI and ARDS. Alongside this suggestion, a preclinical study proposed that AD protects against ALI and ARDS in a mouse model [18].

Augmentation of AD through inhibition of ADA and ADK may enhance the clinical results in Covid-19 patients. This can be attained via its immunosuppressive and anti-inflammatory effects, especially in the late phase, to counteract the exaggerated immune response that usually occurs in this phase of the disease [19, 20]. However, the immunosuppressive effect mediated by AD may affect viral clearance and increase viral replication in the initial phase of infection as ADA controls the negative impact of AD on the immune cells and immune response [21].

It has been reported that ADA inhibitors like pentostatin improves ARDS and its associated chronic inflammatory reactions [22]. Besides, ADK inhibitors like iodotubercidin attenuate ALI and ARDS via inhibition of neutrophil migration and improvement of the lung capillary-alveolar barrier. Nevertheless, the preclinical studies didn't recommend using ADK inhibitors due to the dangerous adverse effects such as liver toxicity and cerebral hemorrhage [23, 24].

Furthermore, AD, through the A2A receptor, activates the regulatory T cell (T_reg_), which regulates the immune response to hypoxia through the reduction of neutrophil infiltrations, cytokine production, and protein extravasations in the lung alveoli [25]. Similarly, AD, through activation of lung peroxisome proliferator-activated receptor gamma (PPARγ), attenuates lung inflammations and interstitial fluid accumulation with the improvement of the alveolar gas exchange [25]. AD, through the A2B receptor, constrains the discharge of the pro-inflammatory cytokines and chemokines in the lung during hypoxia and mechanical ventilation injury [26]. Consequently, through its immune-modulating effect, AD might be an efficient agent in managing Covid-19-induced ARDS. Falcone et al. [27] reported a case with Covid-19 and ARDS treated using standard therapy and oxygen (21%) mixed with AD. They noticed that within one month, there was a dramatic improvement in clinical and radiological outcomes of this patient. Likewise, a retrospective analysis reported by Correale et al. [28] showed that standard therapy and oxygen (21%) mixed with AD could substantially enhance the consequences of 14 Covid-19 patients suffering from ALI. Furthermore, a docking analysis study illustrated that AD has anti-SARS-CoV-2 by interfering with the main viral protease and its inhibition [29, 30]. Therefore, AD has a critical role in managing Covid-19 by suppressing SARS-CoV-2 and associated lung inflammations.

Of note, Covid-19 complications are linked with coagulopathy and thrombosis owing to endothelial dysfunction and platelet activation by SARS-CoV-2 as evident through the elevated D-dimer serum levels [31]. The reduction of AD during SARS-CoV-2 and cytokine storm is due to augmentation of the intracellular transport of AD through ENT-1. The reduction of AD contributes to platelet activation and thrombosis through the reduction of cAMP [32]. DIP inhibits ENT-1 and ENT-2, so the extracellular AD would be increased through attenuation of the intracellular transport. Elevation of the extracellular AD is linked with platelet inhibition via activation of cAMP, thus, it will reduce the risk of intravascular thrombosis [33, 34]. Previously, it has been shown that DIP attenuates ALI in experimental rats through modulation of the lung AD via blocking ENT-1 and ENT-2 pathways [35].

**Fig. 1:**
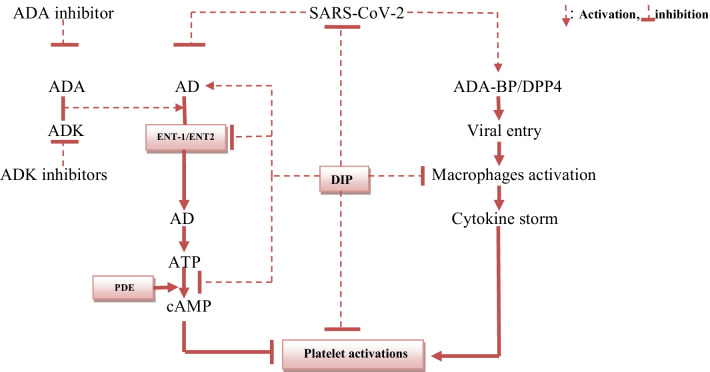
Adenosinergic pathway and the contribution of DIP in Covid-19. DIP inhibits SARS-CoV-2 replication, platelet activation, PDE, ENT-1, and macrophages activation with activation of AD. ADK and ADA metabolize AD. SARS-CoV-2 activates ADA-BP on DPP-4 and inhibits AD

Besides, DIP, through inhibition of PDE3, may prevent coagulopathy and ALI in Covid-19 patients through modulation of platelet function and lung inflammation [34]. The interactions between DIP and AD in SARS-CoV-2 infection might be beneficial, as shown in Fig. 1.

Therefore, DIP, through modulation of the AD/PDE axis, has a critical contribution to the management of Covid-19. DIP inhibits the pro-inflammatory cytokines and lung inflammation during Covid-19. Also, DIP, through escalation of AD, leads to potent anti-inflammatory effects which participate in the reduction of cytokine storm and the associated ALI [36, 37]. Moreover, the important contribution of DIP to Covid-19 is correlated with its wide-spectrum potential antiviral effects [38]. Interestingly, docking studies revealed that DIP blocks SARS-CoV-2 main protease (Mpro), leading to attenuation of Covid-19 pneumonia [39, 40]. Liu et al. [41] showed that when DIP was administered in severely ill Covid-19 patients, it led to a significant clinical improvement with the reduction of D-dimer. Similarly, DIP improves immune recovery and inhibits thrombosis and coagulation disorders in cases having Covid-19 [41].

Into the bargain, activation of nod-like receptor pyrin 3 (NLRP3) inflammasome usually accompanies SARS-CoV-2 infection. This causes the discharge of interleukins (IL-1β and IL-18) and the development of ALI and ARDS. Thus, DIP, by blocking ENT-1 and ENT-2 pathways, may reduce NLRP3 inflammasome-mediated ALI and ARDS [42]. In addition, DIP down-regulates inflammatory signaling pathway such as NF-κB, matrix metalloproteinase (MMP1, MMP9), and cyclooxygenase-2 (COX-2). This can be accomplished by suppressing the macrophage-1 gene (Mac-1) [43], which is enormously activated in Covid-19 infection. It has been proposed that COX-2 inhibitors have a remarkable outcome in the management of Covid-19 by reduction of lung inflammation and IL-6 [44]. Similarly, MMPs inhibitors like aprotinin reduce the pro-inflammatory cytokines-mediated ALI and ARDS [45, 46].

Renin-angiotensin system (RAS) is highly affected in Covid-19 owing to the down-regulation of ACE2 and interconnected with the progress of ALI, ARDS, and injury in multiple organs [47]. It has been reported recently that DIP reduces RAS and circulating AngII serum levels through an AD-dependent pathway [46, 47] or PDE inhibition pathway [48, 49]. Furthermore, DIP also mitigates Covid-19-induced complications like acute kidney injury [50], acute coronary syndrome [51], acute brain injury [52], and cytokine storm-mediated multi-organ injury [53, 54].

**Fig. 2:**
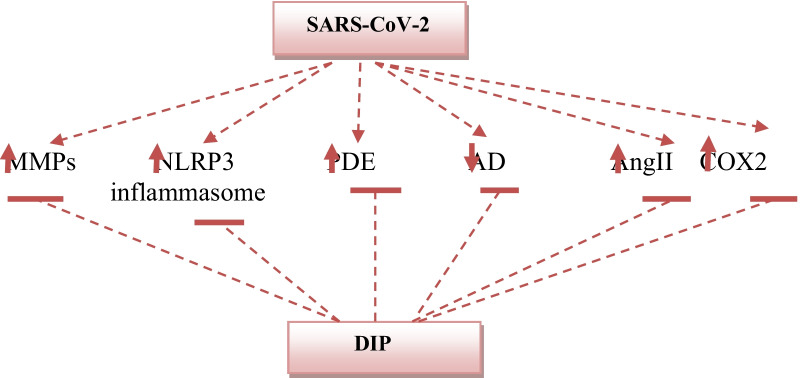
Effects of DIP in Covid-19. Infection with SARS-CoV-2 augments MMPs, COX-2, NLRP3 inflammasome, PDE, and AngII with reduction of AD. DIP inhibits SARS-CoV-2-mediated inflammatory processes

Therefore, this study highlighted that DIP has potential pleiotropic effects (Fig. 2) that mitigate SARS-CoV-2 infection and the accompanying extra-pulmonary disorders. Thereby, DIP might be a "Holy Grail" for Covid-19 patients who are severely ill.

## Conclusion

Hence, DIP has a possible therapeutic impact in the managing of Covid-19. This effect can be achieved either directly, through anti-SARS-CoV-2, anti-inflammatory, and anti-platelets consequences, or indirectly, through augmentation of the extracellular AD, which has immune-regulatory and anti-inflammatory outcomes. However, extensive randomized clinical trials and clinical and prospective studies are necessary to declare the safety and clinical effectiveness of DIP and AD modulators in the management of Covid-19.

## Data Availability

All data are involved in the manuscript.

## Abbreviations

ACE2: Angiotensin-converting enzyme 2
AD: Adenosine
ADA: Adenosine deaminase
ADA-BP: Adenosine deaminase-binding protein
ADK: Adenosine kinase
ADP: Adenosine diphosphate
ALI: Acute lung injury
AMP: Adenosine monophosphate
AngII: Angiotensin II
ARDS: Acute respiratory distress syndrome
ATP: Adenosine triphosphate
COPD: Chronic obstructive pulmonary disease
COX-2: Cyclooxygenase-2
Covid-19: Coronavirus disease 2019
DIP: Dipyridamole
DPP-4: Dipeptidyl dipeptidase 4
ENT: Equilibrative nucleoside transporter
HIV: Human immunodeficiency virus
IL: Interleukins
INF-γ: Interferon-gamma
RAS: Renin-angiotensin system
RBCs: Red blood cells
SARS-CoV-2: Severe acute respiratory coronavirus 2
Treg: Regulatory T cell
MERS-CoV: Middle east respiratory syndrome coronavirus
MMP: Metalloproteinase
NLRP3: Nod-like receptor pyrin 3
PPARγ: Peroxisome proliferator-activated receptor gamma
PDE: Phosphodiesterase
